# Acute renal denervation decreases lactate production in the kidney cortex in experimental septic shock

**DOI:** 10.1186/2197-425X-3-S1-A267

**Published:** 2015-10-01

**Authors:** EH Post, F Su, K Hosokawa, FS Taccone, A Herpain, J Creteur, J-L Vincent, D De Backer

**Affiliations:** Erasme University Hospital, Intensive Care Unit, Brussels, Belgium

## Introduction

The kidney is not considered a source of lactate during septic shock. Renal lactate metabolism, however, is complex with consumption and production occurring simultaneously. As β_2_ stimulation during endotoxemia results in increased lactate production in the muscle [1], lactate formation in the renal cortex may result from sympathetic stimulation as well.

## Objectives

To investigate the effect of acute renal denervation on kidney cortex metabolism in an ovine model of septic shock.

## Methods

After baseline measurements were taken (T-1h), the animals were randomized to bilateral renal denervation (n = 7) or sham procedure (n = 7). Surgical denervation consisted of stripping the renal artery from the adventitia and applying a 20% phenol in 95% alcohol solution. In sham animals, the adventitia was left untouched and the artery was moistened with a 0.9% NaCl-solution. A flow probe was placed around the left renal artery to measure renal blood flow (RBF) and a microdialysis probe was inserted into the kidney cortex to measure interstitial glucose, pyruvate and lactate levels.

Sepsis was induced by injecting 1.5 g/kg of autologous feces into the abdominal cavity (T0h). The animals received fluid (Ringer's lactate and HES 130/0.42 in a 1:1 ratio) to keep the pulmonary artery occluded wedge pressure at baseline levels.

The animals were observed for 18 hours and data were analyzed for main effect of time and interaction between group and time using linear mixed models. In case of significance, pairwise comparisons were carried out using Student's t-test. A p-value of less than 0.05 was considered statistically significant.

## Results

Septic shock was associated with increased cortical lactate levels after 6 hours and increased interstitial pyruvate concentrations after 12 hours (table 2). Renal denervation caused an immediate and temporary increase in RBF but a similar reduction in RBF was observed in both groups after the onset of shock (table 1). However, denervation prevented the late increase in interstitial pyruvate (T12h) and interstitial lactate (T18h) concentrations. A similar reduction in creatinine clearance and urine output (UO) was observed in both groups.

**Table 1:** Table 1

		T-1h	T0h	T6h	T12h	T18h
MAP, mmHg	Control	88 ± 11	85 ± 7	73 ± 21	43 ± 8 *	33 ± 8 *
	Denervation	90 ± 10	85 ± 6	78 ± 9 *	49 ± 9 *	41 ± 13 *
RBF, mL/min	Control	194 ± 48	157 ± 24 *	116 ± 62 *	44 ± 28 *	29 ± 31 *
	Denervation	163 ± 69	237 ± 59 #,*	186 ± 36 #	64 ± 42 *	38 ± 35 *
Creatinine Clearance, mL/min	Control	61 ± 17	61 ± 18	59 ± 42	5 ± 6 *	1 ± 2 *
	Denervation	77 ± 43	54 ± 29	58 ± 32	7 ± 8 *	0 ± 0 *
UO, mL/kg/hr	Control	2.0 ± 1.0	2.2 ± 1.2	1.8 ± 0.9	0.1 ± 0.1 *	0.1 ± 0.1 *
	Denervation	2.5 ± 1.9	3.2 ± 2.9	2.0 ± 1.0	0.4 ± 0.4 *	0.0 ± 0.0 *

*[General hemodynamics and renal function]*

**Table 2:** Table 2

		T0h	T6h	T12h	T18h
Glucose, mg/dL	Control	21 ± 8	22 ± 12	22 ± 13	18 ± 17
	Denervation	23 ± 5	25 ± 6	25 ± 10	12 ± 11
Lactate, mmol/L	Control	0.5 ± 0.3	0.7 ± 0.3	1.5 ± 0.7 *	4.5 ± 1.2 *
	Denervation	0.7 ± 0.3	0.9 ± 0.3	1.3 ± 0.6	2.5 ± 0.8 #,*
Pyruvate, μmol/L	Control	23 ± 13	42 ± 13 *	75 ± 29 *	66 ± 47
	Denervation	30 ± 6	32 ± 6	41 ± 23 #	22 ± 8 #

*[Renal cortex metabolism]*

## Conclusions

Renal denervation attenuated the increase in lactate and pyruvate levels in the kidney cortex, suggesting that the renal nerve regulates aerobic glycolysis in the kidney cortex during sepsis. These metabolic alterations do not appear to have a causal relationship with the observed renal dysfunction.
